# OPTimizing Irradiation through Molecular Assessment of Lymph node (OPTIMAL): a randomized open label trial

**DOI:** 10.1186/s13014-020-01672-7

**Published:** 2020-10-02

**Authors:** Manuel Algara López, Elvira Rodríguez García, Inmaculada Beato Tortajada, Francisco José Martínez Arcelus, Juan Salinas Ramos, José Reyes Rodríguez garrido, Xavier Sanz Latiesas, Ana Soler Rodríguez, Germán Juan Rijo, Amanda Flaquer García

**Affiliations:** 1 Radiation Oncology Department, Del Mar Hospital, Autonomous University of Barcelona, Hospital del Mar Medical Research Institute, Passeig Maritim, 25, 08003 Barcelona, Spain; 2 Radiation Oncology Department, Virgen del Rocío University Hospital, Seville, Spain; 3 Radiation Oncology Department, Castellón Provincial Hospital, Castellón de La Plana, Spain; 4 Radiation Oncology Department, La Fe Polytechnic University Hospital La Fe, Valencia, Spain; 5 Radiation Oncology Department, Santa Lucia General University Hospital, Cartagena, Spain; 6 Radiation Oncology Department, University Hospital of Leon, León, Spain; 7 Radiation Oncology Department, Del Mar Hospital, Pompeu Fabra University, Hospital del Mar Medical Research Institute, Barcelona, Spain; 8 Radiation Oncology Department, De La Ribera Hospital, Alzira, Spain; 9 Radiation Oncology Department, Cabueñes University Hospital, Gijón, Spain; 10 Araba Txagorritxu University Hospital, Vitoria, Spain

**Keywords:** Breast cancer, Conserving surgery, OSNA, Nodal irradiation, Incidental dose

## Abstract

**Background:**

Conservative surgery followed by breast and nodal irradiation is the standard loco-regional early breast cancer (BC) treatment for patients with four or more involved lymph nodes. However, the treatment strategy when fewer nodes are involved remains unclear, especially when lymphadenectomy has not been performed. Sensitive nodal status assessment molecular techniques as the One-Step Nucleic Acid Amplification (OSNA) assay can contribute to the definition and standardization of the treatment strategy. Therefore, the OPTIMAL study aims to demonstrate the feasibility of incidental irradiation of axillary nodes in patients with early-stage BC and limited involvement of the SLN.

**Methods:**

BC patients who underwent conservative surgery and whose SLN total tumour load assessed with OSNA ranged between 250–15,000 copies/µL will be eligible. Patients will be randomized to receive irradiation on the breast, tumour bed, axillary and supraclavicular lymph node areas (intentional arm) or only on the breast and tumour bed (incidental arm). All areas, including the internal mammary chain, will be contoured. The mean, median, D5% and D95% doses received in all volumes will be calculated. The primary endpoint is the non-inferiority of the incidental irradiation of axillary nodes compared to the intentional irradiation in terms of 5-year disease free survival. Secondary endpoints comprise the comparison of acute and chronic toxicity and loco-regional and distant disease recurrence rates.

**Discussion:**

Standardizing the treatment and diagnosis of BC patients with few nodes affected is crucial due to the lack of consensus. Hence, the quantitative score for the metastatic burden of SLN provided by OSNA can contribute by improving the discrimination of which BC patients with limited nodal involvement can benefit from incidental radiation as an adjuvant treatment strategy.

**Trial registration:**

ClinicalTrial.gov, NCT02335957; https://clinicaltrials.gov/ct2/show/NCT02335957

## Background

It has been shown that breast and nodal irradiation reduce mortality and disease recurrence in breast cancer (BC) patients [1]. Therefore, there is evidence supporting the use of adjuvant radiotherapy after conservative surgery. Since nodal irradiation increases survival rates, it is recommended in patients with four or more involved lymph nodes (LN) [2], as well as in patients with other types of locally advanced tumours with LN involvement [3]. A randomized clinical trial carried out by the British Columbia Cancer Agency, showed that the 20-year disease-free survival (DFS) of patients subjected to the combination of chemotherapy and radiation was 13% higher than that of the patients who were treated with chemotherapy alone [4]. Similar results were achieved in the DBCG 82 b&c trial showing a difference of 10% in the 15-year overall survival rate between the treatment combination and chemotherapy alone (39% vs. 29%, respectively) [5]. Furthermore, a study funded by the Canadian Cancer Society concluded that adjuvant radiation treatment of limited LN-positive patients (0–3 involved nodes) led to a decrease in disease recurrence [6]. However, there is no consensus on the recommendation of radiotherapy when the nodal involvement ranges between 1 and 3 LN [7].

Furthermore, this situation became even more unclear since Giuliano et al. demonstrated no significant benefit in loco-regional control with completion of axillary lymph node dissection (ALND) in comparison to no ALND in patients with 1–2 involved LN [8, 9]. On the other hand, AMAROS [10] and OTOASOR [11] trials demonstrated that nodal irradiation should be regarded as the recommended treatment for patients with few involved LN, instead of ALND. In line with their results, the Canadian trial NCIC-CTG MA20 [6] showed that local irradiation combined with regional irradiation improved the DFS as well as the loco-regional and distant control of the disease in high-risk patients with negative and with positive LN, mostly with 1 to 3 involved LN, while Poortmans et al. [12] demonstrated that the irradiation of the regional nodes in patients with limited axillary disease results in an increase of the DFS. Darby et al. [13] reported that, after breast-conserving surgery, the application of radiotherapy to the breast reduced mortality and the disease recurrence by half. Finally, a systematic review, which included more than 20,000 patients from 45 studies, concluded that breast irradiation reduced the loco-regional relapse even in patients without LN involvement [14]. Later on, the meta-analysis conducted by Budach et al. [15, 16] concluded that additional regional radiation to the internal mammary and medial supraclavicular LN improved overall and disease-free survival rates in stage I-III BC patients.

The uncertainty about the proper radiation therapy entails the need for a method that standardizes the choice of treatment and complements the limited diagnostic information [17–19], such as nodal involvement assessment. The OSNA (One-Step Nucleic Acid Amplification) assay provides a quantitative value of the metastatic burden of the sentinel lymph node (SLN) by measuring the mRNA expression of the tumour marker cytokeratin 19 (CK19) [20]. The OSNA assay not only provides automated and complete intraoperative analyses of the SLN but also standardized and reliable results for SLN metastatic status. The total tumour load (TTL), defined as the sum of the CK19 mRNA copies from all positive SLNs of the patient, entails a quantitative score that integrates both the metastatic burden and the number of involved SLN. The TTL score was proved to be an independent predictor factor of the axillary nodal status, where only 14.7% of patients with TTL beneath 15.000 copies/µl had other positive non-SLN [21]. Recently, the PLUTTO study results also proved its impact on the prognosis of BC patients [22].

Therefore, the OPTIMAL study (OPTimizing Irradiation through Molecular Assessment of Lymph node) aims to demonstrate the non-inferiority of incidental irradiation of axillary nodes in comparison to intentional irradiation in terms of the 5-year DFS of patients with early-stage BC and limited involvement of the SLN according to the OSNA quantitative score.

## Methods/design

### Study design

**Fig. 1:**
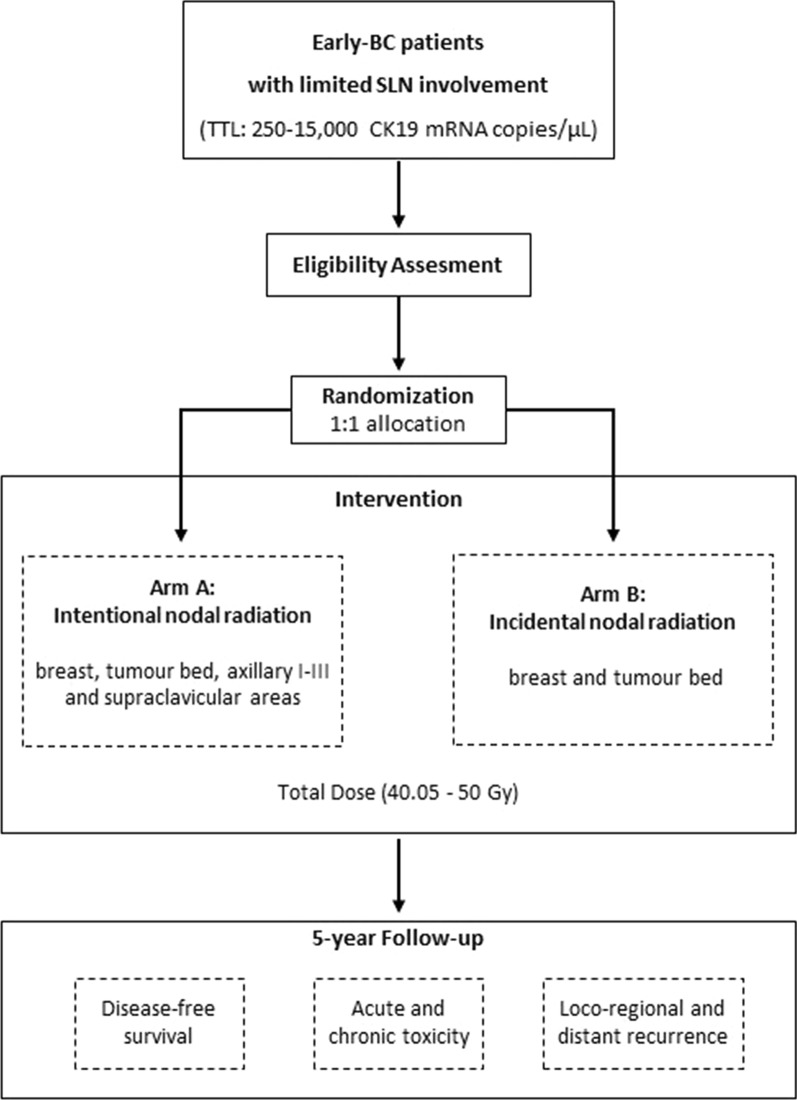
OPTIMAL-I study design. *BC* breast cancer, *CK19* cytokeratin 19, *mRNA* messenger ribonucleic acid, *SLN* sentinel lymph node, *TTL* total tumour load

The OPTIMAL study is an open-label multicentre and international trial (NCT02335957) conducted in over 40 sites in Spain, Portugal, and Italy. Eligible patients will be randomized with a 1:1 allocation ratio to receive irradiation on breast, tumour bed, axillary and supraclavicular lymph node areas (intentional arm) or only on breast and tumour bed (incidental arm), as depicted in Fig. 1. Randomization by blocking within centres to minimize imbalance of treatments among centres will be performed using the online randomization software RANDI2 (www.dkfz.de; Heidelberg University) embedded in the electronic case record form (eCRF). All patients will have to provide their Informed Consent prior to the inclusion in the study.

### Eligibility criteria

#### Inclusion criteria

- Older than 18 years old
- Female invasive ductal breast cancer patients
- T1–T2 tumour stage
- Previous treatment with breast-conserving surgery without axillary lymphadenectomy
- OSNA assayed SLN with a TTL within the 250–15,000 copies/µL range
- Karnofsky Performance scale Index ≥ 70%
- Written and signed Informed Consent

#### Exclusion criteria

- Invasive lobular carcinoma and other histologic subtypes
- Bilateral breast cancer
- Male breast cancer patients
- Patients who underwent a mastectomy or ipsilateral dissection of axillary LN
- Having received previous thoracic irradiation
- Systemic neoadjuvant therapy prior to surgery
- Contraindications to radiotherapy such as pregnancy or serious collagen disease.
- Other neoplasms and or any associated sever comorbidities that may interfere with the study

### Endpoints

#### Primary endpoint

The primary endpoint is the non-inferiority of incidental irradiation of axillary nodes in contrast to intentional irradiation, in terms of the 5-year DFS of patients diagnosed with early-stage breast cancer with limited involvement of the SLN treated with breast-conservative surgery without axillary lymphadenectomy.

#### Secondary endpoints

The secondary endpoints established were the following:

- Loco-regional tumour recurrence in the two treatment arms within the 5-year follow-up period.
- Distant tumour recurrence in the two treatment arms within the 5-year follow-up period.
- Acute toxicity induced by either the incidental or intentional radiation treatment.
- Chronic toxicity induced by either the incidental or intentional radiation treatment.
- Total irradiation dose (Gy) received in axillary levels I-III, supraclavicular fossa, and internal mammary chain volumes.

### Radiation procedure

#### Volume contouring

All node areas from axillary levels I-III, the supraclavicular fossa and the internal mammary chain must be contoured in all patients regardless of the assigned arm following the guidelines of the Radiation Therapy Oncology Group [23]. The Clinical Target Volumes (CTV) will be delimited as stated below:

- *Breast* The CTV includes the whole breast’s soft tissues ranging from 5 mm below the skin surface to the deep fascia, excluding the muscle and underlying ribs. The posterior margin should not extend beyond the deep fascia or the edges of the visible/palpable breast in medial and lateral directions.
- *Tumour bed* The delineation of the tumour bed is only mandatory for patients who require a boost. It is strongly recommended to delimit the medial, lateral, superior, inferior, anterior and posterior margins of the surgical cavity at the time of the surgery, with clips or gold seeds. In the absence of implanted fiducial markers, the tumour bed may be localised if there is a well-defined seroma, considering visible changes in the computed tomography (CT) or by means of support from previous mammograms or magnetic resonance imaging. This volume shall be defined by drawing around the implanted markers and changes in the surrounding tissue architecture.
- *Supraclavicular* In the incidental arm, nodes will be contoured as part of the procedure whereas, in the control group, this will be hidden so as not to influence the dosimetric planning. The limits will be cranial edge (thyroid cartilage); caudal edge (clavicle head); medial edge (1 cm lateral to the lateral wall of the trachea excluding the thyroid); lateral edge (acromioclavicular joint); anterior edge (the sternocleidomastoid muscle and the clavicle); posterior edge (the trapezius muscle).
- *Axilla level III* In the incidental arm, nodes will be contoured as part of the procedure whereas, in the control group, this will be hidden to not influence the dosimetric planning. The limits will be the cranial edge (5 mm cranial to the axillary vessels); caudal edge (1 cm caudal to the axillary vessels); medial edge (the ribcage); lateral edge (the res major muscle); anterior edge (the pectoralis major muscle); posterior edge (chest wall and intercostal muscles).
- *Axilla level II* In the incidental arm, nodes will be contoured as part of the procedure whereas, in the control group, this will be hidden to not influence the dosimetric planning. The limits will be the cranial edge (5 mm cranial to the axillary vessels); caudal edge (caudal edge of pectoralis minor muscle); medial edge (the medial border of the pectoralis minor muscle); lateral edge (the lateral border of the pectoralis minor muscle); anterior edge (the anterior surface of the pectoralis minor muscle); posterior edge (chest wall and intercostal muscles).
- *Axilla level I* In the incidental arm, nodes will be contoured as part of the procedure whereas, in the control group, this will be hidden to not influence the dosimetric planning. The limits will be the cranial edge (1 cm below caput humeri); caudal edge (free edge of pectoralis major muscle); medial edge (the lateral border of the pectoralis minor muscle); lateral edge (the medial edge of the dorsolateral muscle); anterior edge (the plane defined by the anterior surface of the pectoralis major and minor lateral dorsal); posterior edge (the anterior surface of the subscapularis muscle).
- *Internal mammary chain* Nodes will be contoured in both arms and will be hidden to not influence the dosimetric planning. The limits will be the cranial edge (cranial edge of rib 1); caudal edge: (cranial edge of rib 5); medial edge (the edge of the sternum); lateral edge (5 mm lateral of internal mammary vessels or 2 cm from the edge of the sternum); anterior edge (dorsal surface of m. pectoralis major, dorsal surface of the sternum); posterior edge (pleura).
- *Organs at risk* The delineation of ipsilateral lung and heart is mandatory for the calculation of dose-volume histograms. The ipsilateral lung must be outlined as a single structure, and care should be taken not to include any airways; the CT must cover the entire lung volume. The heart must be outlined as a single structure to the extent of the pericardial sac; the major blood vessels (superior and inferior) are excluded and the superior extent may be simplified by identifying the vessels superior to the heart. Contralateral breast, contralateral lung, oesophagus, caput humeri, thyroid and spinal cord may be delineated according to institutional protocols.

Enlarging all volumes by adding a 5–10 mm margin in all directions to create the planning treatment volume (PTV) is recommended.

#### Treatment planning

The treatment planning must be carried out on a 3D system with tissue heterogeneity correction and matrix resolution of 2.5 mm. Techniques in the supine position are allowed and the prone position is not allowed.

- *Breast* Tangent beam pair arrangement to encompass the whole breast is recommended, intending to minimize the total dose in the ipsilateral lung and heart. The treatment plan can be optimized with any dose compensation system, (virtual edges, mechanical wedges, automatic wedges, field-in-field IMRT and steep and shoot or sliding windows IMRT). The treatment will be performed with photons of 6 MV–15 MV. The isocenter will be located within the breast PTV in the experimental group and may be located outside the PTV if the single isocenter technique is being used in the control group. To minimize irradiated ipsilateral lung and heart volumes, collimator rotation and shield with multileaf collimator is allowed.
- *Tumour bed* Photons or electrons are allowed. Mini-tangential photon fields or single electron field (also mixed energies) can be utilised. The use of boluses can be considered if needed. Simultaneous integrated boost brachytherapy or intraoperative irradiation is also allowed if the dose contribution to the nodal areas can be calculated. The bolus can be used. The dose and fractionation choice is left at the discretion of the treating physician.
- *Node areas* In the control group, the treatment planning must be optimized to ensure that the nodal areas receive the prescribed dose (except for the internal mammary chain), minimizing the dose at the organs at risk. High tangent, AP/PA, conformational 3D and IMRT techniques are allowed. In the experimental group, treatment planning for nodal areas is not performed. The internal mammary chain will not be irradiated intentionally in any case.

An unplanned gap of up 3 days is acceptable. Longer non-planned interruptions should be compensated by hyperfractionation of the daily normal dose.

#### Dose prescription

All doses prescribed will follow the International Commission on Radiation Units and Measurements guidelines. A minimum of 95% of the volume must receive at least 95% of the prescribed dose. Less than 5% of the volume may receive a dose of 105% and less than 2% should receive a dose of 107%, with a maximum overall dose of 110%. The dose in the breast must be 50.0 Gy by 25 fractions of 2.0 Gy in 5 weeks or through hypofractionated schedules as 40.05 Gy in 15 fractions of 2.67 Gy for 3 weeks. In the tumour bed, the schedule and total dose are left to the centre’s criteria. In node areas, the intentional irradiation to the breast and tumour bed will be calculated. Limiting doses for the organs at risk must be: (1) ipsilateral lung: V20 less than 25%; and (2) heart: V20 less than 10% and V40 less than 5%.

#### Radiotherapy verification

Verification methods will be conducted in both arms. Treatment verification is required at the first treatment fraction and allowed on the three first fractions. The verification must be performed using electronic portal images of the treatment beam; either with MV or kV. Orthogonal images or cone-beam images can be used on the verification of the isocentre. Weekly control will be performed, and systematic daily control is also allowed.

### Follow-up

Patients will be followed for up to 5 years after the intervention according to the visit schedule detailed in Table 1. In each visit, a physical examination and recurrence assessment will be performed. An image assessment will be requested every year after the intervention. Reasons for discontinuing follow-up must be reported.

**Table 1:** Schedule of visits and assessments (dots) that will be performed during the 5-year follow-up period

Post-intervention (year)	1					2		3		4		5	
Post-intervention (month)	1	3	6	9	12	18	24	30	36	42	48	54	60
Acute toxicity	●												
Physical exam	●	●	●	●	●	●	●	●	●	●	●	●	●
Image evaluation of local recurrence					●		●		●		●		●
Survival and disease recurrence	●	●	●	●	●	●	●	●	●	●	●	●	●
Chronic toxicity	(Continuous recording)												
Co-medication, (adjuvant)	(Continuous recording)												

### Data collection and analysis

#### Data collection

The study data will be recorded in an eCRF (OpenClinica®, LLC). The demographic and clinical data requested is depicted in Table 2. Acute and chronic toxicity will be recorded according to the Common Terminology Criteria for Adverse Events v4.0 (CTCAE) [24] criteria. Specific follow-up outcomes will be assessed and registered during the visits scheduled as stated in Table 1.

**Table 2:** Data collected in the electronic case record form

Panel in eCRF	Data recorded
Informed consent	Date
I/E criteria	Yes; not
Demographic data	Age at inclusion; Menstrual State
Comorbidities	If yes: specify
Cancer Histology and Receptors, SLN OSNA	Tumour grade; Tumour size (maximum diameter); P53 (%); Ki67 (%); Lymphovascular infiltration; Ductal Ca in situ; % Estrogenic receptors; % Progesterone receptors; HER2 receptor status; OSNA TTL of SLN
Previous medication (Adjuvant therapy)	Drug; Start date; Stop date
Type of surgery	Tumour surgery; date; Margins
Randomization	Treatment randomly allocated; Randomization date
Radiotherapy intervention	Patient completed the allocated treatment (If no: main reason); Start date; End date; Treatment gaps (If yes, reason); Dose per volume (Mean; Median; D95; D5; Volume) in the breast, tumour bed, supraclavicular and axillary levels I-III, and Internal mammary chain
Co-medication	Drug; Start date; Stop date
Survival and disease recurrence	Date of follow-up visit (If not performed, reason); Local recurrence; Regional recurrence (If yes: nodal level); Distant recurrence (If yes: organ); Vital status (if dead: date and cause)
Image evaluation of local recurrence	Technique; Local recurrence (if yes: Maximum diameter)
Physical examination^a^	Palpable breast tumour (if yes: size, skin infiltration, inflammatory carcinoma, satellite lesions); Palpable axillary nodes (if yes: size); Palpable supraclavicular nodes (if yes: size); Node staging
Acute and chronic toxicity	CTCAE term; Grade; Start date; Stop date; Status (recovered w/o sequels; death)

*eCRF* electronic case record form, *OSNA* One-Step Nucleic Acid Amplification, *TTL* total tumour load, *SLN* sentinel lymph node, *CTCAE* Common Terminology Criteria for Adverse Events

^a^Physical examination is conducted at follow-up visits. It is also addressed at baseline and radiotherapy sessions to confirm the compliance of eligible criteria. If suspicious LNs are detected, the patient will discontinue the study

#### Sample size estimation

A total of 1.400 patients must be recruited to show the non-inferiority of the experimental arm (incidental irradiation) with an 80% of statistical power when we assume a 5-year recurrence rate of 15% in the control arm (intentional irradiation) [8], a 5% non-inferiority margin, a yearly dropout rate of 5%, and a fixed sample design. Despite the short follow-up period, the large number of patients to be included in the study will preserve the statistical significance of the survival rates and will be enough to evaluate differences in the toxicity rates.

#### Statistical analysis

Two sets of patients will be analysed: the intention-to-treat (ITT) group, which includes all randomized patients, to describe the baseline clinic-pathological patients’ characteristics; and the per-protocol subset, which includes patients who finish the intervention treatment as planned with all dosimetry data completed, for the endpoint. A descriptive analysis will be carried out reporting absolute and relative frequencies for all variables recorded and stratified by treatment group. Two analyses of the primary endpoint (disease-free survival rate) will be conducted. The confirmatory analyses will be carried out using a non-inferiority long-rank test in the ITT set of patients, which includes all randomized patients regardless of whether the treatment or follow-up are accomplished. A secondary explanatory analysis will be conducted in the per-protocol subgroup, which will include patients who will have finished the intervention treatment with all dosimetry data completed, by an adjusted Cox regression model using the covariates: centre, age at inclusion, tumour size, hormone receptor status, Her2 receptor status, and OSNA results. With regards to the secondary endpoints: the outcomes of loco-regional and distant recurrence will be analysed using the Cox approach, this time in the ITT set. Acute toxicity will be analysed by a chi-squared test comparing frequencies in both treatment groups. Chronic toxicity will be analysed by using Kaplan Meier curves and comparing them by a standard log-rank test. Interim analyses are planned when 85 events and 169 events will be reached.

#### Current status of the trial

From February 2015 to February 2020, a total of 451 patients have been recruited (224 in the intentional arm and 227 in the incidental arm). Currently, acute toxicity events have been reported in 319 cases. A total of 48 chronic toxicity and 13 recurrence events have been also informed.

### Discussion

Nowadays, early BC patients with 2 or fewer involved LN undergo nodal irradiation instead of lymphadenectomy although it is unclear whether these patients require local-specific treatment. This uncertainty arises from that current studies do not clearly describe the radiation volumes used as adjuvant treatment. Furthermore, some physicians advocate that the breast of these patients should be treated with high or modified tangents [25]. Therefore, although it is not stated as LN irradiation, the axillary levels I and II are being irradiated. Accordingly, more than 70% of patients who had not undergone lymphadenectomy received LN radiation and even 19% received unallowed supraclavicular irradiation in the study conducted by Giuliano et al. [26]. In line with previous research, a recent larger meta-analysis reported that expanding the radiation field to the axillary and supraclavicular nodes after ALND reduces locoregional recurrences without an improvement on the overall and cancer-specific survival [27]. Thus, patients’ survival would be improved due to the inclusion of the internal mammary chain in the radiation plan. Consequently, elucidating the effect of node radiation by means incidental or intentional doses in early BC is needed.

Thus, the OPTIMAL trial was designed to outline an evidence-based strategy for the treatment of BC patients with limited nodal involvement by determining the non-inferiority of incidental radiation of the axillary nodes in comparison with intentional radiation. The ESTRO (European Society for Radiotherapy and Oncology) meeting in Assisi stated the importance of further investigations on regional lymph node treatment and highlighted the design and expected results of the OPTIMAL, SENOMAC, and POSNOC studies, all of them focused on the treatment of patients with limited axillary disease [28].

Final results from the INSEMA and SOUND trials [29, 30], which mainly questioned the use of no-ALND instead of SLN biopsy procedures in early BC, may also contribute to better outline treatment strategies in early BC. Nonetheless, even though patients with limited LN involvement (1–3 macrometastases) are considered in these studies, whole-breast radiation therapy is conducted.

In the OPTIMAL trial, the SLN status and the limited LN involvement is determined according to the OSNA quantitative molecular assay [20]. At the time of the study design, few studies with regards to OSNA performance had been reported. Nowadays, its contribution to the improvement of BC patient staging and prognosis has been widely reported [31]. In fact, LN assessment with OSNA assay and the evaluation of the TTL as a quantitative score of the metastatic burden of the patients is recommended for the management of BC patients in the SESPM (Spanish Society of Senology and Breast Pathology) and the NICE (National Institute for Health and Care Excellence) guidelines [32–34]. This technique provides quick and standardized results at in-house diagnostic laboratories, which prompts the patient’s diagnosis and treatment tailoring since central laboratories are not required for a reliable LN assessment.

In conclusion, due to the lack of consensus on the proper therapeutic strategy of BC patients with few involved LNs standardizing the treatment and diagnosis is crucial. Hence, the quantitative score for metastatic burden provided by OSNA assay can contribute by improving the discrimination of the BC patients with a limited nodal involvement who can benefit from incidental radiation as an adjuvant treatment strategy.

## Data Availability

The datasets generated and/or analysed during the current study are not publicly available due to confidentiality reasons but are available from the corresponding author on reasonable request.

## Abbreviations

ALND: Axillary lymph node dissection
AP/PA: Anterior-posterior/posterior-anterior
BC: Breast cancer
CK19: Cytokeratin 19
CTCAE: Common Terminology Criteria for Adverse Events
DFS: Disease-free survival
eCRF: Electronic case report form
ESTRO: European Society for Radiotherapy and Oncology
ITT: Intention-to-treat
LN: Lymph node
mRNA: Messenger ribonucleic acid
NICE: National Institute for Health and Care Excellence
OSNA: One-Step Nucleic Acid Amplification
SESPM: Spanish Society of Senology and Breast Pathology
SLN: Sentinel lymph node
TTL: Total tumour load
